# USP39 regulates the cell cycle, survival, and growth of human leukemia cells

**DOI:** 10.1042/BSR20190040

**Published:** 2019-04-05

**Authors:** Chunxia Liu, Xiaojian Yao, Ming Li, Yaming Xi, Li Zhao

**Affiliations:** Department of Hematology, the First Hospital of Lanzhou University, Lanzhou 730000, P.R. China

**Keywords:** Apoptosis, Cell proliferation, Cell cycle, IRF1, Leukemia, USP39

## Abstract

Ubiquitin-specific peptidase 39 (USP39) is one member of the cysteine proteases of the USP family, which represents the largest group of DeUbiquitinases with more than 50 members in humans. The roles of USP39 in human cancer have been widely investigated. However, the roles of USP39 in human leukemia and the underlying mechanism remain unknown. Here we reported the function of USP39 in human leukemia. We observed that the expression of *USP39* was up-regulated in human leukemia cells and the high expression of *USP39* was correlated with poor survival of the patients with leukemia. Lentivirus-mediated knockdown of *USP39* repressed the proliferation and colony formation of human leukemia cell lines HL-60 and Jurkat cells. Mechanism study showed that *USP39* knockdown induced the arrest of cell cycle and apoptosis of leukemia cells. In addition, our microarray and bioinformatic analysis demonstrated that USP39 regulated diverse cellular signaling pathways that were involved in tumor biology, and several pivotal genes (*IRF1, Caspase 8*, and *SP1*) have been validated by quantitative real-time polymerase chain reaction. Knockdown or *IRF1* partially restored the proliferation rate of leukemia cells with *USP39* knockdown. Taken together, our findings implicate that USP39 promotes the development of human leukemia by regulating cell cycle, survival, and proliferation of the cells.

## Introduction

Leukemia is a group of cancers that usually begin in the bone marrow and result in high numbers of abnormal white blood cells. These white blood cells are not fully developed and are called blasts or leukemia cells [1]. Our understanding of leukemia biology has been radically transformed over recent years with a more realistic grasp of its multilayered cellular and genetic complexity [2]. Leukemogenesis requires enhanced self-renewal, which is induced by oncogenes [3]. However, the underlying molecular mechanisms of leukemia remain incompletely understood.

The cysteine proteases of the USP family represent the largest group of DeUbiquitinases, with more than 50 members in humans. The deubiquitinase ubiquitin-specific peptidase 39 (USP39) is an essential splicing factor. USP39 is essential for mitotic spindle checkpoint integrity and controls mRNA-levels of *Aurora B* [4]. High expression of USP39 is associated with the development of vascular remodeling [5].

The roles of USP39 in human cancer have been widely investigated. For instance, USP39 promotes colorectal cancer growth and metastasis through the Wnt/β-catenin pathway [6]. USP39 deubiquitinase is essential for *KRAS proto-oncogene, GTPase* (*KRAS*) oncogene-driven cancer [7]. Additionally, USP39 regulates the growth of hepatocellular carcinoma via Forkhead box M1 (FoxM1) [8,9]. Moreover, overexpression of *USP39* predicts poor prognosis and promotes tumorigenesis of prostate cancer via promoting epidermal growth factor receptor (*EGFR*) mRNA maturation and transcription elongation [10]. However, the roles of USP39 in human leukemia remain unknown.

Here in the present work, we aimed to elucidate the function of USP39 in human leukemia. We observed that USP39 was overexpressed in human leukemia, which was correlated with the survival of patients. Molecular, cellular and bioinformatic analysis demonstrated that USP39 regulated the growth, cell cycle, and survival of leukemia cells.

## Materials and methods

### Patients

Peripheral blood samples were collected from acute myelocytic leukemia (AML) patients or transplant donors from 2010 to 2015 at the First Hospital of Lanzhou University. Mononuclear cells were isolated from diagnostic peripheral blood of 21 adult patients with AML. Mononuclear cells from healthy individuals were taken as controls. For further RNA analysis, the CD34^+^ cells were selected using immunomagnetic columns (Miltenyi Biotec) with CD34 antibody (R&D, #MAB72271) as described previously [11]. Written informed consent was obtained from all the patients and participants. The present study was approved by the Ethics Committee of The First Hospital of Lanzhou University. The present study was conducted in accordance with the Declaration of Helsinki and written informed consent was obtained from the participant.

### Quantitative real-time PCR

Freshly isolated cells and cultured cells were subjected to RNA isolation with TRIzol (Invitrogen). One ug of total RNA was then used for cDNA synthesis with the SuperScript™ III CellsDirect™ cDNA Synthesis System (ThermoFisher). Next, quantitative real-time polymerase chain reaction (qRT-PCR) was performed with the QuantiTect SYBR^®^ Green PCR Kit (QIAGEN) to determine the relative expression of target genes. Glyceraldehyde-3-phosphate dehydrogenase (GAPDH) was used as housekeeping gene. The following primers were used in the present study. The relative expression of USP39 was normalized to GAPDH and analyzed using comparative delta cycle threshold (CT) method (CT^USP39^ − CT^GAPDH^). A lower CT value represents a higher relative expression of USP39.

*GAPDH* forward 5′-GGAGCGAGATCCCTCCAAAAT-3′
*GAPDH* reverse 5′-GGCTGTTGTCATACTTCTCATGG-3′
*USP39* forward 5′-GGTTTGAAGTCTCACGCCTAC-3′
*USP39* reverse 5′-GGCAGTAAAACTTGAGGGTGT-3′
*IRF1* forward 5′-ATGCCCATCACTCGGATGC-3′
*IRF1* reverse 5′-CCCTGCTTTGTATCGGCCTG-3′
*Caspase 8* forward 5′-GTTGTGTGGGGTAATGACAATCT-3′
*Caspase 8* reverse 5′-TCAAAGGTCGTGGTCAAAGCC-3′
*SP1* forward 5′-GTGGCCGCTACCTTCACTG-3′
*SP1* reverse 5′-GCCCCACTCCTACTTGGTC-3′

### Western blot

Total proteins were extracted from cultured cells with RIPA lysis buffer (Thermo) supplied with protease inhibitor cocktail (Roche). 40 ug of total protein was subjected to sodium dodecyl sulfate polyacrylamide gel electrophoresis (SDS-PAGE) separation and Western blot with the standard protocol [12]. The following primary antibodies were used in the present study: anti-GAPDH (Cell Signaling Technology), anti-USP39 (Abcam), anti-H3K27ac (Cell Signaling Technology), anti-H3K27me3 (Cell Signaling Technology), and anti-IRF1 (Cell Signaling Technology). The secondary antibodies were purchased from Invitrogen. The immune-activity was detected using ECL-Plus kit (Amersham Biosciences).

### Cell lines and cell culture

Human leukemia cell lines Jurkat, HL-60, and K-562 were obtained from ATCC. The normal bone marrow cell line (HS-5) were purchased from the American Type Culture Collection. The bone marrow cell line and leukemia cells were cultured in alpha-minimal essential medium (ThermoFisher). HEK293T cells were cultured in Rosewell Park Memorial Institute 1640 (ThermoFisher). All culture medium was supplied with 10% fetal bovine serum (ThermoFisher), 100 units/ml penicillin and streptomycin (Gibco). The cells were cultured at 37 °C and 5% CO_2_. To analyze the proliferation rate of the cells, cells were seeded at 1 × 10^4^ or 1 × 10^3^ cells/ml in 10-cm dishes and the cell number was counted every day.

### Lentivirus package, infection, and transduction

In the present study, lentivirus-mediated short hairpin RNAs (shRNAs) were used to knock down the expression of *USP39* in leukemia cells. Control shRNA or sh*USP39* were cloned into the pLKO.1 plasmid (Addgene). The shRNA sequences targeting human *USP39* (NM_001256728.1) is 5′-GCTCCAGGACTCCCTCAATAA-3′ and the shRNA sequences targeting human *IRF1* (NM_001354924.1) is 5′-GGAAATTACCTGAGGACATCAAAG-3′. To prepare lentivirus, we transfected HEK293T cells with the lentivirus particles, psPAX2, and pVSVG in according to the manufacturer (Life Technologies). For transduction, virus-containing supernatant was collected and the leukemia cells were incubated with the supernatant for 48 h, then the cells were selected with puromycin (1 μg/ml) for an additional 48 h.

### Cell proliferation assay

Leukemia cells were transduced with sh*USP39* or control shRNA. Then the cells were subjected to proliferation assay. Cell number was counted with CCK-8 kit (Byeotime) in according to the manufacturer’s protocol.

### Methylcellulose colony-forming cell assay

The methylcellulose colony-forming cell assay was performed as described previously [13]. In all, 0.9 ml of 1 × 10^3^ cells/ml were combined with 1.2 ml of 2.1% (w/v) methylcellulose and 0.9 ml fetal bovine serum; 3 ml was plated in triplicate on 35 mm plates with gridlines. Plates were imaged and counted after 9 days at 37 °C in 5% CO_2_ with the EVOS XL Core Imaging System (Life Technologies).

### Cell cycle analysis

Leukemia cells were infected with lentivirus carrying shCtrl or sh*USP39* for 24 h. Cell cycle progression was determined by propidium iodide (PI) staining using a flow cytometer. Briefly, cells were fixed with 70% cold ethanol at 4°C overnight, washed twice with ice-cold PBS, and incubated with 10 mg/ml RNase at 37°C. Cell cycle was monitored by using PI staining of nuclei. PI uptake was analyzed by fluorescence-activated cell sorting on flow cytometry (FACSCalibur, Becton Dickinson).

### Apoptosis analysis

The cells were infected with control or sh*USP39* lentivirus for 24 h. Then, the Annexin V-FITC Apoptosis Detection Kit (Becton Dickinson) was applied to analyze the apoptosis of leukemia cells according to the manufacturer’s protocol. The data were analyzed with FACSCalibur flow cytometer.

### Microarray

Total RNA from HL-60 cells was extracted using Trizol reagent (Invitrogen). NanoDrop 2000 and Agilent Bioanalyzer 2100 were used to detect the RNA quantity and quality. Affymetrix human GeneChipprimeview was used for microarray processing to determine a gene expression profile according to the manufacturer’s instructions. Significantly different genes between HL-60 cell treated with sh*USP39* and shCtrl were identified depending on the following criteria: *P*<0.05 and the absolute fold change >2. The biofunction and pathway enrichment analysis were performed using IPA®Software (http://www.ingenuity.com).

### Statistical analysis

The values are expressed as the mean ± SEM of three independent repeats if no other information is indicated. Student’s *t* test was applied to analyze the difference between two groups. *P* values less than 0.05 were considered significant. The statistical analysis was performed with the software GraphPad Prism 7 and SPSS 20.1.

## Results

### High expression of USP39 predicts poor survival of patients with leukemia

**Figure 1:**
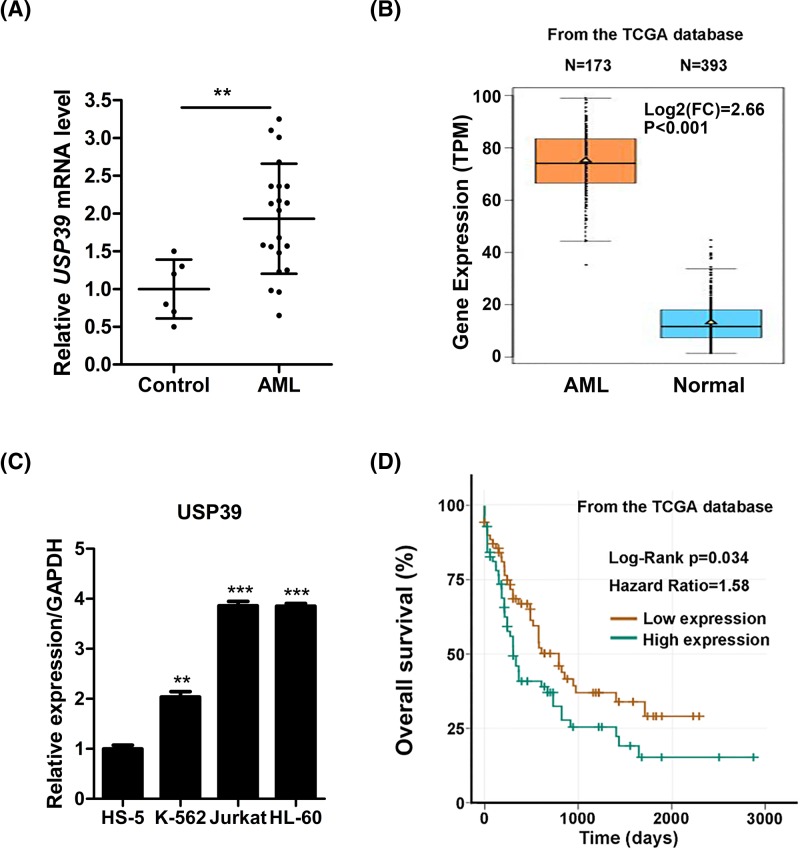
Expression of *USP39* is associated with leukemia (**A**) Quantitative real-time PCR results showing that *USP39* mRNA level was overexpressed in human leukemia. **P<0.01. *n* = 6 in control group and *n* = 21 in leukemia group. (**B**) Gene expression data from TCGA database showing that *USP39* mRNA level was overexpressed in human leukemia. (**C**) Quantitative real-time PCR results showing the relative expression of *USP39* mRNA in leukemia cell lines Jurkat, HL-60, and K-562 as well as a normal bone marrow cell line (HS-5). **P<0.01 and ***P<0.001 *vs.* HS-5. (**D**) Data from the TCGA database showing high *USP39* expression are correlated with poor survival of patients with leukemia.

The functions of the *USP39* in human leukemia remains unknown. To explore the potential roles of *USP39* in human leukemia, we first examined the expression of *USP39* in human leukemia cells. We collected leukemia cells from 21 patients with leukemia and analyzed the expression of *USP39.* The results showed that *USP39* mRNA level was significantly up-regulated in leukemia cells isolated from leukemia patients compared with that from control donors (Figure 1A). In addition, we also analyzed the expression pattern of *USP39* in leukemia samples using the The Cancer Genome Atlas (TCGA) database (https://cancergenome.nih.gov/). The results also showed that the expression of *USP39* was increased in leukemia samples (Figure 1B). We also tested the expression content of *USP39* in three leukemia cell lines (HL-60, Jurkat, K562). The relative *Ct* value indicated that *USP39* was expressed at high level in leukemia cell lines (Figure 1C). Since the high expression of *USP39* in human leukemia, we next investigated whether *USP39* expression level was correlated with survival using the TGCA database. The results showed that high expression of *USP39* in leukemia cells was correlated with poor survival of the patients (Figure 1D). Taken together, these findings demonstrated that *USP39* was overexpressed in human leukemia cells and high expression of *USP39* predicted poor survival.

### *USP39* regulates the growth of leukemia cells

**Figure 2:**
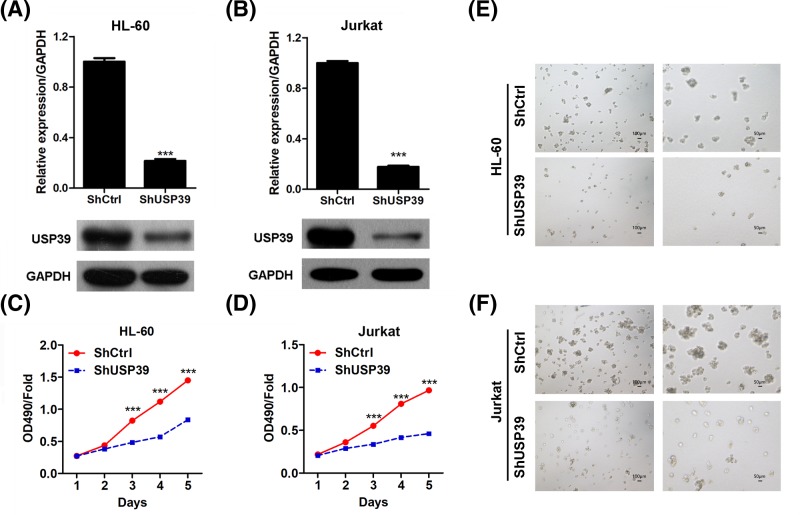
Knockdown of the expression of *USP39* represses the growth of leukemia cells (**A**) qRT-PCR and Western blot results showing the results of *USP39* knockdown in HL-60 cells. HL-60 cells were infected with lentivirus expressing short-hairpin RNA (shRNA) targeting *USP39* or control shRNA for 48 h, then the cells were subjected to qRT-PCR and Western blot assays. ***P<0.001 *vs.* shCtrl. (**B**) qRT-PCR and Western blot results showing the results of *USP39* knockdown in Jurkat cells. Jurkat cells were infected with lentivirus expressing shRNA targeting *USP39* or control shRNA for 48 h, then the cells were subjected to qRT-PCR and Western blot assays. ***P<0.001 *vs.* shCtrl. (**C**) *USP39* knockdown represses the proliferation of HL-60 cells. HL-60 cells were infected with lentivirus expressing sh*USP39* or control shRNA, then the cell numbers were monitored at the indicated time point. ***P<0.001 *vs.* shCtrl. (**D**) *USP39* knockdown represses the proliferation of Jurkat cells. Jurkat cells were infected with lentivirus expressing sh*USP39* or control shRNA, then the cell numbers were monitored at the indicated time point. ***P<0.001 *vs.* shCtrl. (**E**) Representative images showing *USP39* knockdown represses colony formation of HL-60 cells. HL-60 cells were transduced with sh*USP39* or control shRNA. Then the cells were subjected to colony formation assay. (**F**) Representative images showing *USP39* knockdown represses colony formation of Jurkat cells. Jurkat cells were transduced with sh*USP39* or control shRNA. Then the cells were subjected to colony formation assay.

Since the high expression of *USP39* in human leukemia, we next aimed to investigate the roles of *USP39* in regulating the cellular behavior of leukemia cells. To this end, we designed lentivirus-mediated short-hairpin RNA targeting *USP3*9 (sh*USP39*). Our qRT-PCR and Western blot results showed that *USP39* expression was significantly knocked down in leukemia cell lines HL-60 and Jurkat cells (Figure 2A,B). Then we prepared leukemia cells with/without sh*USP39* transduction and cell proliferation assay was performed. The results showed that *USP39* knockdown markedly repressed the proliferation rate of HL-60 and Jurkat cells since day 3 (Figure 2C,D). Colony formation is a key feature of stem cells and cancer cells [14]. We next investigated the effects of *USP39* on the colony formation capacity of leukemia cells. Significantly, our results showed that *USP39* knockdown reduced both the number and size of clones formed by HL-60 or Jurkat cells (Figure 2E,F). Taken together, these findings demonstrated that USP39 knockdown inhibited the growth of leukemia cells *in vitro.*

### *USP39* regulates the cell cycle in leukemia cells

**Figure 3:**
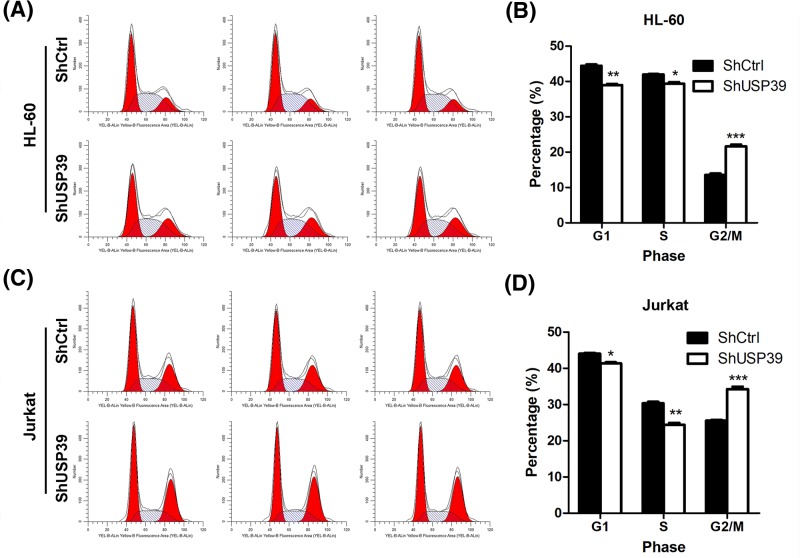
*USP39* knockdown induces cell cycle arrest (**A**) Representative results showing *USP39* knockdown induces cell cycle arrest in HL-60 cells. HL-60 cells were infected with lentivirus expressing sh*USP39* or control shRNA for 24 h, then the cells were subjected to cell cycle analysis. (**B**) Quantitative results of the cell cycle phase of results in (**A**). *P<0.05, **P<0.01, ***P<0.001 *vs.* shCtrl. (**C**) Representative results showing *USP39* knockdown induces cell cycle arrest in Jurkat cells. Jurkat cells were infected with lentivirus expressing sh*USP39* or control shRNA for 24 h, then the cells were subjected to cell cycle analysis. (**D**) Quantitative results of the cell cycle phase of results in (**C**). *P<0.05, **P<0.01, ***P<0.001 *vs.* shCtrl.

We next explored whether cell cycle arrest contributed to the effects of *USP39* on the growth of leukemia cells. We, therefore, infected HL-60 and Jurkat cells with lentivirus carrying sh*USP39* or control shRNA, and then the cultured cells were subjected to analysis of cell cycle with FACS. The results showed that *USP39* knockdown decreased the percentage of cells in G1 and S phases, while the percentage of cells in G2/M phase was increased in HL-60 cells. This observation indicated that the cell cycle was blocked at the G2/M phase by *USP39* knockdown in HL-60 cells (Figure 3A,B). Similarly, we observed that *USP39* knockdown induced cell cycle arrest at G2/M phase in Jurkat cells (Figure 3C,D). Collectively, these findings demonstrated that USP39 regulates cell cycle of leukemia cells.

### *USP39* regulates apoptosis in leukemia cells.

**Figure 4:**
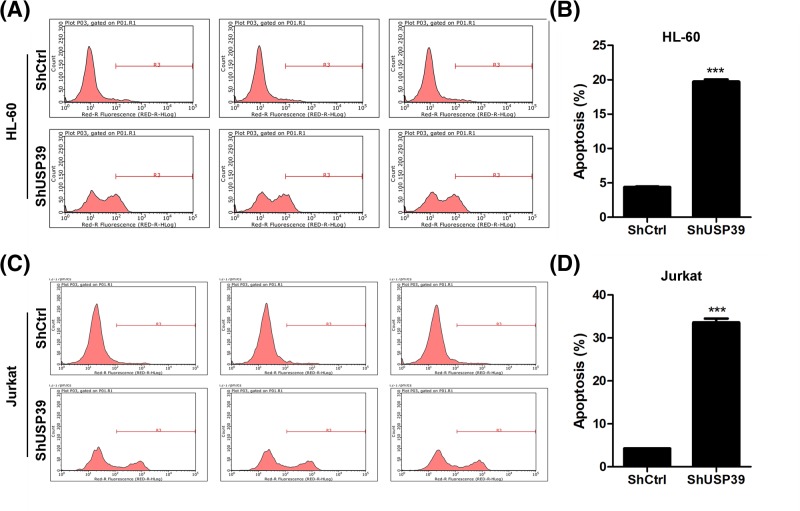
*USP39* knockdown induces apoptosis of leukemia cells (**A**) Representative results showing *USP39* knockdown induces apoptosis in HL-60 cells. HL-60 cells were infected with lentivirus expressing sh*USP39* or control shRNA for 24 h, then the cells were subjected to analyze the apoptosis. (**B**) Quantitative results of the apoptosis in (**A**). ***P<0.001 *vs.* shCtrl. (**C**) Representative results showing *USP39* knockdown induces apoptosis in Jurkat cells. Jurkat cells were infected with lentivirus expressing sh*USP39* or control shRNA for 24 h, then the cells were subjected to analyze the apoptosis. (**D**) Quantitative results of the apoptosis in (**C**). ***P<0.001 *vs.* shCtrl.

Resistance to apoptosis is another feature of cancer cells. We also investigated the effects of *USP39* knockdown on the apoptosis of leukemia cells. The leukemia cells HL-60 and Jurkat cells were infected with lentivirus carrying sh*USP39* or control shRNA. FACS assay was performed to analyze the percentage of apoptotic cells. The results showed that *USP39* knockdown increased 4-fold of apoptosis in HL-60 cells, and 6–7-fold in Jurkat cells (Figure 4A**–**D). Therefore, *USP39* also controls the survival of leukemia cells.

### Bioinformatic analysis of *USP39* downstream genes.

**Figure 5:**
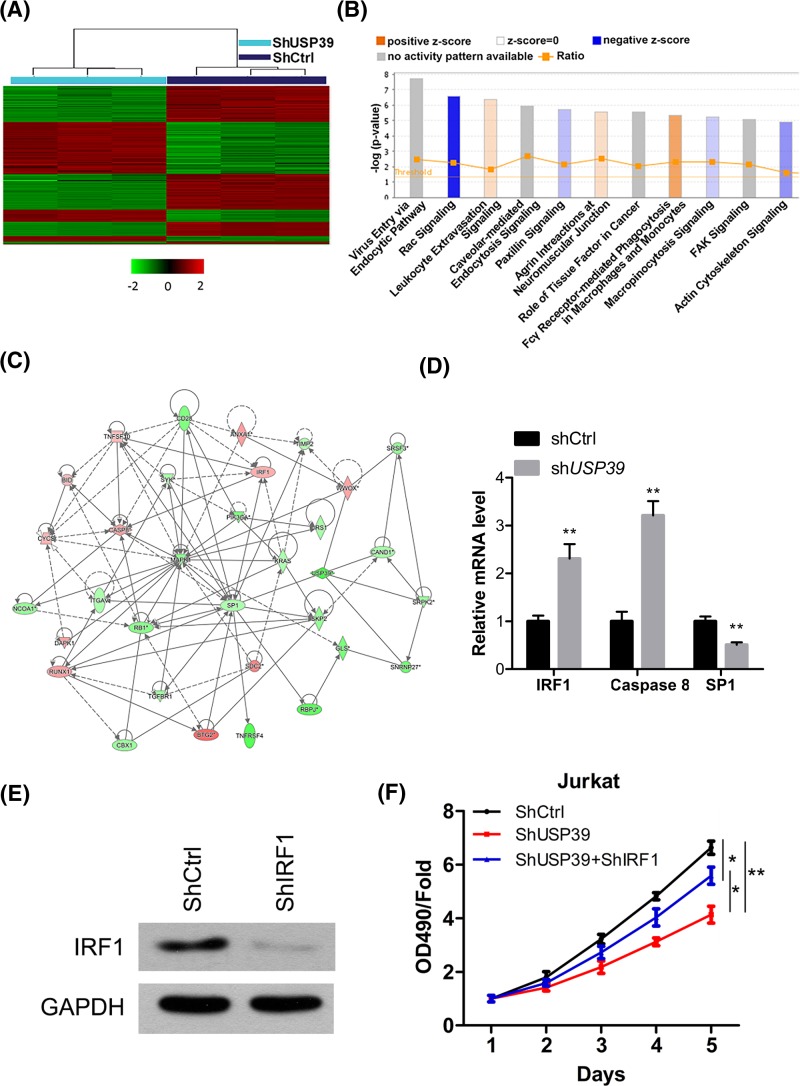
Bioinformatic analysis of USP39 downstream genes (**A**) Heatmap showing differentially expressed genes in HL-60 cells with or without *USP39* knockdown. HL-60 cells were infected with lentivirus carrying sh*USP39* or shCtrl for 48 hours. Then the cells were subjected to microarray for analysis of differentially expressed genes. (Criteria: *P*<0.05, absolute fold change >2). (**B**) Functional pathway enrichment of differential genes was analyzed based on IPA analysis. (**C**) The interactional network was constructed between *USP39* and genes involved in IPA pathway cell cycle. Green circles represent downregulated genes, red circles represent up-regulated and genes of gray circles represent no expression changing. (**D**) USP39 regulates the expression of *IRF1, Caspase 8* and *SP1*. HL-60 cells were infected with shCtrl or sh*USP39* for 48 hours and then western blot was performed to analyze the expression of *IRF1, Caspase 8* and *SP1.* **P<0.01 *vs.* shCtrl. (**E**) Western blot results showing the results of *IRF1* knockdown in HL-60 cells. HL-60 cells were infected with lentivirus expressing shRNA targeting *IRF1* or control shRNA for 48 hours, then the cells were subjected to western blot assay. (**F**) *IRF1* knockdown restores the proliferation rate of HL-60 cells with *USP39* knockdown. HL-60 cells were infected with lentivirus expressing indicated shRNA and subjected to cell proliferation assay. *P<0.05, **P<0.001 by one-way ANOVA analysis.

The above results showed that *USP39* was critical for the survival and growth of human leukemia cells. However, the mechanisms underlying *USP39-*mediated phenotypes and the downstream pathways were still unknown. Therefore, HL-60 cells infected with lentivirus expressing sh*USP39* or control shRNA were subjected to microarray assay to the global gene expression profile of these cells. We identified and 1638 genes showing with significantly significant difference differential expression were identified (absolute fold change>2, and *P*<0.05), including 724 up-regulated genes and 914 down-regulated genes (Figure 5A). The IPA pathway analysis was performed and the differentially expressed genes were enriched in 11 pathways, including leukocyte extravasation, tissue factor in cancer (Figure 5B). The gene-interacting network analysis showed that *USP39* was correlated with diverse genes involved in survival, proliferation or apoptosis (Figure 5C). To confirm these results, we selected three key genes involved in leukemia (*interferon-regulatory factor 1 [IRF1], Caspase 8*, and *specificity protein 1 [SP1]*) to confirm the gene expression of those factors was regulated by USP39. As expected, knockdown of *USP39* significantly affected the mRNA level of *IRF1, Caspase 8* and *SP1* (Figure 5D). However, the effects of USP39 on the expression of the targets did not rely on epigenetic modification because USP39 knockdown did not affect the level of H3K27ac and H3K27me3, two histone marks of transcriptional activation (Supplementary Figure S1). We next explored whether the targets of *USP39* was involved in the function of *USP39*. Therefore, we knocked down the expression of *IRF1* with lentivirus-mediated shRNA in HL-60 cells (Figure 5E). The cell proliferation assay showed that *IRF1* knockdown partially restored the proliferation rate of HL-60 cells with *USP39* knockdown (Figure 5F). These findings demonstrated that *IRF1* partially contributed to the function of *USP39* in regulation of the growth of leukemia cells.

## Discussion

In the present work, we identify *USP39* as a regulator of human leukemia. The expression of *USP39* mRNA level was significantly up-regulated in the leukemia cells compared with those from the controls. Our loss-of-function experiments demonstrated that knockdown of the expression of *USP39* repressed the proliferation of leukemia cells, induced cell cycle arrest, and cell apoptosis. Furthermore, we performed a microarray assay and found that *USP39* regulated the expression of diverse genes in human leukemia cells, including *IRF1, Caspase 8*, and *SP1*, which were validated by qRT-PCR experiments.

The USP family members are essentially involved in the development of human leukemia. small-molecule inhibitors of USP1 promote the degradation of inhibitor of DNA binding 1 (ID1), and are cytotoxic to leukemic cells [15]. USP7 cooperates with NOTCH1 to drive the oncogenic transcriptional program in T cell leukemia [16]. USP7 inhibition alters homologous recombination repair and targets chronic lymphocytic leukemia CLL cells independently of ATM/p53 functional status [17]. Overexpression of USP44 induces chromosomal instability and is frequently observed in human T-cell leukemia [18]. Deubiquitinase USP48 promotes ATRA-induced granulocytic differentiation of acute promyelocytic leukemia cells [19]. The roles of other USP members in human leukemia remain unknown.

USP39 is a member of the USP family. Previous reports demonstrated that USP39 was significantly involved in diverse types of cancer. USP39 regulates the growth of hepatocellular carcinoma cells via regulating the transcriptional factor FoxM1 [8]. In patients with prostate cancer, high expression of USP39 predicts poor prognosis and USP39 promotes tumorigenesis of prostate cancer cells via promoting *EGFR* mRNA maturation and transcription elongation [10]. In addition, as a target of microRNA-133a, USP39 promotes progression of pancreatic cancer via the AKT pathway [20]. In the present study, we identified the roles of USP39 in human leukemia. We observed that the expression of USP39 was significantly up-regulated in human leukemia and high expression of USP39 in human leukemia cells predicted poor overall survival.

*USP39* regulates the growth of SMMC-7721 cells [8]. Knockdown of *USP39* by lentivirus-mediated RNA interference suppresses the growth of oral squamous cell carcinoma [8]. Indeed, *USP39* also controlled the growth of leukemia cells. We observed that lentivirus-mediated knockdown of *USP39* significantly repressed the proliferation rate of leukemia cells. In addition, the knockdown of USP39 also reduced the number and size of clones formed by leukemia cells, implicating that the colony formation of leukemia cells was controlled by *USP39.*

USP39 also controls the cell cycle and apoptosis of leukemia cells. We showed that *USP39* knockdown induced the cell cycle arrest at G2/M phase in two lines of leukemia cells. In addition, USP39 knockdown significantly induced apoptosis of leukemia cells. *USP39* is essential for mitotic spindle checkpoint integrity and controls mRNA levels of *Aurora B* [4]. Down-regulation of *USP39* suppresses the proliferation and induces the apoptosis of human colorectal cancer cells [21]. Knockdown of *USP39* inhibited the growth of osteosarcoma cells and induced apoptosis *in vitro* [22]. Indeed, knockdown of *USP39* induces cell cycle arrest and apoptosis in melanoma [23]. Therefore, *USP39* is a strong regulator for cell cycle and apoptosis in diverse types of cancer. Further study is needed to explore the certain protein targets of *USP39* that are involved in controlling cell cycle and apoptosis

Bioinformatic analysis revealed that *USP39* significantly modified the transcriptional profile of leukemia cells. The IPA pathway analysis showed that *USP39* regulated diverse pathways that were involved in cancer, including Rac signaling, leukemia extravasation signaling, tissue factor in cancer, focal adhesion kinase (FAK) signaling. The expression gene network analysis showed that *USP39* knockdown significantly regulated a downstream network involving *IRF1, Caspase8*, and *SP1. IRF1, Caspase 8* and *SP1* are important regulators for human leukemia. Our qRT-PCR data also validated the regulation of these genes by *USP39.* Importantly, we demonstrated that IRF1 partially contributed to the function of USP39 in leukemia cells. In addition, we analyzed whether USP39 regulated the expression of the target genes through regulating the epigenetic modification. However, we did not observe the effects of USP39 on H3K27ac or H3K27me3. Therefore, USP39 may regulate the expression of the targets through other mechanism.

Our findings suggest and USP39 may serve as prognostic biomarker and therapeutic target. However, since it is one of the members in a huge family of similar proteins it may be easy to acquire resistance after a period of time and there may be toxicity issues with the therapy targeting these proteins. Further works are needed to explore the mechanism by which USP39 regulate the targets and whether USP39 could serve as a therapeutic target.

In conclusion, we identify *USP39* as an oncogene-like protein in human leukemia. *USP39* controls the proliferation, cell cycle, and apoptosis of leukemia cells. Therefore, *USP39* may serve as a potential target for the treatment of human leukemia.

## Supporting information

**Supplementary Figure 1:** Effects of USP39 knockdown on histone modification.

## Abbreviations

AML: acute myelocytic leukemia
ATCC: American Type Culture Collection
CT: cycle threshold
EGFR: epidermal growth factor receptor
GAPDH: glyceraldehyde-3-phosphate dehydrogenase
IRF1: interferon regulatory factor 1
PCR: polymerase chain reaction
PI: propidium iodide
SDS-PAGE: sodium dodecyl sulfate polyacrylamide gel electrophoresis
shRNA: short hairpin RNA
SP1: Sp1 transcription factor
TCGA: The Cancer Genome Atlas
USP39: ubiquitin-specific peptidase 39

## References

[1] Hourigan C.S., Gale R.P., Gormley N.J., Ossenkoppele G.J. and Walter R.B. (2017) Measurable residual disease testing in acute myeloid leukaemia. Leukemia 31, 1482–1490 10.1038/leu.2017.113 28386105

[2] Greaves M. (2016) Leukaemia ‘firsts’ in cancer research and treatment. Nat. Rev. Cancer 16, 163–172 10.1038/nrc.2016.3 26911190

[3] Zhou F., Liu Y., Rohde C., Pauli C., Gerloff D., Kohn M. (2017) AML1-ETO requires enhanced C/D box snoRNA/RNP formation to induce self-renewal and leukaemia. Nat. Cell Biol. 19, 844–855 10.1038/ncb3563 28650479

[4] van Leuken R.J., Luna-Vargas M.P., Sixma T.K., Wolthuis R.M. and Medema R.H. (2008) Usp39 is essential for mitotic spindle checkpoint integrity and controls mRNA-levels of aurora B. Cell Cycle 7, 2710–2719 10.4161/cc.7.17.6553 18728397

[5] He S., Zhong W., Yin L., Wang Y., Qiu Z. and Song G. (2017) High expression of ubiquitin-specific peptidase 39 is associated with the development of vascular remodeling. Mol. Med. Rep. 15, 2567–2573 10.3892/mmr.2017.6297 28447728 PMC5428656

[6] Yuan X., Sun X., Shi X., Wang H., Wu G., Jiang C. (2017) USP39 promotes colorectal cancer growth and metastasis through the Wnt/beta-catenin pathway. Oncol. Rep. 37, 2398–2404 10.3892/or.2017.5454 28259917

[7] Fraile J.M., Manchado E., Lujambio A., Quesada V., Campos-Iglesias D., Webb T.R. (2017) USP39 deubiquitinase is essential for KRAS oncogene-driven cancer. J. Biol. Chem. 292, 4164–4175 10.1074/jbc.M116.762757 28154181 PMC5354494

[8] Yuan X., Sun X., Shi X., Jiang C., Yu D., Zhang W. (2017) USP39 regulates the growth of SMMC-7721 cells via FoxM1. Exp. Therapeutic Med. 13, 1506–1513 10.3892/etm.2017.4115 PMC5377580 28413501

[9] Pan Z., Pan H., Zhang J., Yang Y., Liu H., Yang Y. (2015) Lentivirus mediated silencing of ubiquitin specific peptidase 39 inhibits cell proliferation of human hepatocellular carcinoma cells in vitro. Biol. Res. 48, 18 10.1186/s40659-015-0006-y 25889525 PMC4389921

[10] Huang Y., Pan X.W., Li L., Chen L., Liu X., Lu J.L. (2016) Overexpression of USP39 predicts poor prognosis and promotes tumorigenesis of prostate cancer via promoting EGFR mRNA maturation and transcription elongation. Oncotarget 7, 22016–22030 26959883 10.18632/oncotarget.7882 PMC5008341

[11] Li L., Osdal T., Ho Y., Chun S., McDonald T., Agarwal P. (2014) SIRT1 activation by a c-MYC oncogenic network promotes the maintenance and drug resistance of human FLT3-ITD acute myeloid leukemia stem cells. Cell Stem Cell 15, 431–446 10.1016/j.stem.2014.08.001 25280219 PMC4305398

[12] Li N., He Y., Wang L., Mo C., Zhang J., Zhang W. (2011) D-galactose induces necroptotic cell death in neuroblastoma cell lines. J. Cell. Biochem. 112, 3834–3844 10.1002/jcb.23314 21826710

[13] Dzneladze I., He R., Woolley J.F., Son M.H., Sharobim M.H., Greenberg S.A. (2015) INPP4B overexpression is associated with poor clinical outcome and therapy resistance in acute myeloid leukemia. Leukemia 29, 1485–1495 10.1038/leu.2015.51 25736236

[14] Hanahan D. and Weinberg R.A. (2011) Hallmarks of cancer: the next generation. Cell 144, 646–674 10.1016/j.cell.2011.02.013 21376230

[15] Mistry H., Hsieh G., Buhrlage S.J., Huang M., Park E., Cuny G.D. (2013) Small-molecule inhibitors of USP1 target ID1 degradation in leukemic cells. Mol. Cancer Ther. 12, 2651–2662 10.1158/1535-7163.MCT-13-0103-T 24130053 PMC4089878

[16] Jin Q., Martinez C.A., Arcipowski K.M., Zhu Y., Gutierrez-Diaz B.T., Wang K.K. (2019) USP7 cooperates with NOTCH1 to drive the oncogenic transcriptional program in T cell leukemia. Clin. Cancer Res. 25 (1), 222–239 30224337 10.1158/1078-0432.CCR-18-1740 PMC6320313

[17] Agathanggelou A., Smith E., Davies N.J., Kwok M., Zlatanou A., Oldreive C.E. (2017) USP7 inhibition alters homologous recombination repair and targets CLL cells independently of ATM/p53 functional status. Blood 130, 156–166 10.1182/blood-2016-12-758219 28495793

[18] Zhang Y., van Deursen J. and Galardy P.J. (2011) Overexpression of ubiquitin specific protease 44 (USP44) induces chromosomal instability and is frequently observed in human T-cell leukemia. PLoS One 6, e23389 10.1371/journal.pone.0023389 21853124 PMC3154946

[19] Li L., Wang Y., Zhang X., Song G., Guo Q., Zhang Z. (2018) Deubiquitinase USP48 promotes ATRA-induced granulocytic differentiation of acute promyelocytic leukemia cells. Int. J. Oncol. 53, 895–903 29901102 10.3892/ijo.2018.4440

[20] Cai J., Liu T., Huang P., Yan W., Guo C., Xiong L. (2017) USP39, a direct target of microRNA-133a, promotes progression of pancreatic cancer via the AKT pathway. Biochem. Biophys. Res. Commun. 486, 184–190 10.1016/j.bbrc.2017.03.025 28286270

[21] Xing Z., Sun F., He W., Wang Z., Song X. and Zhang F. (2018) Downregulation of ubiquitin-specific peptidase 39 suppresses the proliferation and induces the apoptosis of human colorectal cancer cells. Oncol. Lett. 15, 5443–5450 29556295 10.3892/ol.2018.8061 PMC5844003

[22] Gan Z., Han K., Lin S., Hu H., Shen Z. and Min D. (2017) Knockdown of ubiquitin-specific peptidase 39 inhibited the growth of osteosarcoma cells and induced apoptosis in vitro. Biol. Res. 50, 15 10.1186/s40659-017-0121-z 28403900 PMC5389082

[23] Zhao Y., Zhang B., Lei Y., Sun J., Zhang Y., Yang S. (2016) Knockdown of USP39 induces cell cycle arrest and apoptosis in melanoma. Tumour Biol. 37, 13167–13176 10.1007/s13277-016-5212-x 27456357

