# Supplementary material for: USP39 regulates the cell cycle, survival, and growth of human leukemia cells
Source: Biosci Rep. 2019 Apr 5;39(4):BSR20190040. doi: 10.1042/BSR20190040 (PMC6449567; doi:10.1042/BSR20190040)
Supplement: Supplementary file 1 [file bsr-39-bsr20190040_Supp1.pdf]

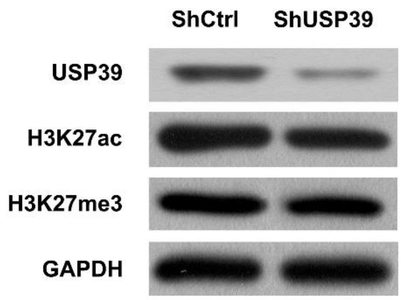

Supplementary Figure 1 Effects of USP39 knockdown on histone modification. HL-60 cells were infected with lentivirus expressing short-hairpin RNA (shRNA) targeting *USP39* or control shRNA for 48 hours, then the cells were subjected to western blot assay.
